# Frontline providers’ perspectives on ageing in place: negotiating older adults’ self-responsibility, preventive services and community support

**DOI:** 10.1186/s12875-026-03404-4

**Published:** 2026-06-02

**Authors:** Linda Aimée Hartford Kvæl, Jardar Sørvoll

**Affiliations:** 1 Health Services Research Unit (HØKH), Akershus University Hospital, P.O. Box 1000, Lørenskog, 1478 Norway; 2 Research Centre for Habilitation and Rehabilitation Models and Services (CHARM), Institute of Health and Society, Faculty of Medicine, University of Oslo, Oslo, Norway; 3 Norwegian Social Research – NOVA, Department of Ageing Research and Housing Studies, Oslo Metropolitan University, St. Olavs Plass, Oslo, NO-0130 Norway

**Keywords:** Ageing in place, Primary care, Municipal health and social care, Frontline providers, Prioritisation, Preventive services, Inclusive communities

## Abstract

**Background:**

Ageing in place implies that older adults remain in their own homes while maintaining autonomy and identity. Municipal health and social care services, which constitute the core of community-based primary care for older adults in Norway, are central to implementing ageing-in-place policies, yet frontline providers face resource constraints, fragmented services, and growing demands that shape how policies are translated into practice. This study explored frontline primary care providers’ perspectives on how municipal health and social care services can support ageing in place and developed practice-based insights into how older adults’ self-responsibility, preventive services and community support are negotiated in everyday work.

**Methods:**

A qualitative interpretive design informed by critical realism and Lipsky’s street-level bureaucracy theory was employed. Eight focus groups were conducted with 38 frontline providers (nurses, nursing assistants, occupational and physical therapists, social workers, case managers, and a physician) across four Norwegian municipalities/urban districts with diverse profiles. Data were analysed using reflexive thematic analysis.

**Results:**

Three main themes were identified: (i) Defining responsibility: supporting ageing preparation and managing expectations – providers described a growing gap between expectations and available resources, increased reliance on families, and the need for clearer communication and ageing-preparation counselling, with task shifting and selective use of welfare technology as ways to manage demand; (ii) Cultivating a culture of prevention: early detection, independence, and rehabilitation – despite policy emphasis on prevention, practice remained largely reactive, with limited protected time for intervention, falls prevention, and rehabilitation; (iii) Building inclusive communities: housing facilities, accessibility, and collaboration – providers underscored the importance of accessible housing, home adaptations, transport, and better coordination of social activities and volunteers to reduce isolation and support autonomy.

**Conclusion:**

Frontline providers described how tight budgets and municipal autonomy favour reactive, legally mandated services over preventive and social support. They pointed to needs for operationalised prioritisation (e.g. simple decision tools), protected time for preventive work, selective use of technology and task shifting, early home adaptations, reliable transport, and navigation and social support. Cross-sector collaboration and sustained support to carers and frontline staff are essential to ensure equitable access and maintain older adults’ independence and dignity at home.

**Supplementary Information:**

The online version contains supplementary material available at 10.1186/s12875-026-03404-4.

## Introduction

Ageing in place has become a major policy priority in Norway and other Western countries as populations age, life expectancy rises, and social and healthcare needs change [1]. While often promoted as a cost-effective approach to long-term care, it is also highly valued by older adults because it supports continuity of identity and autonomy [2]. At the same time, ageing in place can exacerbate social inequalities [3]. Older adults and their families differ in their preparedness and resources for ageing-related planning, sometimes referred to as “independence literacy” [4]. Research points to five key determinants of successful ageing in place: the physical environment (home and community), formal and informal care, social networks, technology, and individual capacity [5]. In the Norwegian context, municipal health and social care services constitute the core of community-based primary care for older adults, encompassing home nursing, rehabilitation, and social support services delivered close to where people live. Throughout this article, ageing in place is therefore understood broadly, drawing on Rogers and colleagues’ (2020) definition of “one’s journey to maintain independence in one’s place of residence as well as to participate in one’s community” [6].

In line with this understanding, recent reforms in Norway have emphasised the importance of living safely at home with a focus on quality of life [7–9]. These reforms mirror broader European goals such as strengthening community-based care, encouraging informal caregivers, reducing costs, and improving access to high-quality services [10]. For older adults, these developments place primary care at the centre of efforts to deliver coordinated, person-centred support across health and social care boundaries. To guide resource allocation in municipal health and care services, Norway has introduced prioritisation criteria focusing on benefit, cost-effectiveness, severity, and autonomy, highlighting individuals’ right to make independent decisions about their own lives [11, 12]. These principles seek to balance gains in health and well-being against resource use and to prioritise those with the greatest needs, yet research shows that applying them consistently in municipal practice remains challenging [13]. Understanding how frontline professionals navigate these challenges is essential to improving the implementation of ageing-in-place policies at the community level.

Michael Lipsky’s (1980) theory of street-level bureaucracy highlights the role of frontline workers, such as healthcare professionals, social workers, and other municipal employees, who interact directly with citizens to implement public policies like ageing in place [14]. Operating under constrained resources, heavy workloads, and institutional pressures, these workers must exercise discretion in their daily decision-making to determine who receives what services, how much, and under what conditions, even within strict legal frameworks [15]. Their choices significantly shape how policies are prioritised and experienced by older adults and relatives, making them key actors in translating policy into practice. In Norway, municipal healthcare professionals act in this capacity when implementing ageing-in-place policies, navigating limited resources and growing care demands. Their decisions influence the accessibility, quality, and equity of services for older adults wishing to remain at home [14]. From a primary care perspective, this street-level discretion mediates how national policies on prevention, rehabilitation and ageing in place are realised—or diluted—in everyday home- and community-based services.

These implementation challenges are compounded as older adults live longer with more complex health conditions, while municipalities face funding and staff shortages [16–18]. Municipal health and social care services also struggle with fragmentation and limited capacity to meet diverse needs [19], including challenges in integrating medical, rehabilitative and social support within primary care teams and coordinating services across sectors and professions. These pressures require frontline staff to allocate resources and make difficult prioritisation decisions in their everyday practice [20]. The aim of this study was therefore to explore street-level frontline providers’ perspectives on how municipal health and social care services can support ageing in place and to develop practice-based knowledge on key issues in their accounts, including older adults’ self-responsibility, preventive services and community support. Given their proximity to the needs of older adults and their central role in the practical implementation of care, the perspectives of these professionals are essential when evaluating and improving policies related to ageing in place [21, 22].

## Methods

Critical realism served as the philosophical framework, acknowledging an objective reality shaped by individual perspectives and social contexts while guiding the development of practical recommendations for addressing complex challenges [23, 24]. In this study, it was used to examine the relationship between frontline professionals’ experiences, the events of their daily work, and the underlying mechanisms that shaped these situations.

### Design

This study used a qualitative interpretive design with focus group interviews to explore healthcare professionals’ shared experiences and diverse perspectives on ageing in place [25]. The study focused on frontline primary care providers working in municipal health and social care services, including home care, rehabilitation, social care, and allocation offices.

### Study setting

In Norway, health and social care services are mainly publicly funded and managed by 357 self-governing municipalities, many of which use a purchase-provider model separating service allocation from provision [26]. The ageing in place approach is a key focus of Norway’s health policy, emphasising home-based services, prevention, and technology to address the challenges of an ageing population [7]. At the municipal level, these services form the backbone of community-based primary care for older adults, including home nursing, practical assistance, rehabilitation, and social support. The study included one medium-sized and one rural municipality, and two urban districts from one of Norway’s largest municipalities, located across three counties in the southern, central, and eastern regions (Table 1). Delivering services over long distances is the main challenge in the municipality with the smallest population and the largest geographical area. The urban districts are densely populated but differ socioeconomically: the western district has higher education levels, social capital, and living standards, than the eastern district, a district that has a higher proportion of residents with minority backgrounds. Together, these differences highlight the challenges municipalities face in ensuring equitable service access and meeting diverse needs among older adults, and illustrate how community-based primary care operates under different demographic, geographic, and socioeconomic conditions within the same national policy.

**Table 1:** Overview of municipalities and districts

Region	County	Type	Population	Area (km²)
Region A	Central	Medium-sized municipality	$\:\approx\:$30,000	700
Region B	South	Rural municipality	< 2,000	1,100
Region C1	East	Urban district, Oslo West	$\:\approx\:$60,000	10
Region C2	East	Urban district, Oslo East	$\:\approx\:$30,000	10

### Participants

We conducted focus group interviews in each municipality/district with a total of 38 municipal employees from diverse professional backgrounds, all of whom were frontline providers working with older adults in daily care (34 women and 4 men). The average age of participants was 42 years (range 22–64 years). The participants had an average of 8 years of experience in their current positions (range 1–27 years), and 9 held higher education at the master’s level. Participating healthcare professionals were distributed across the four Norwegian settings: 7 participants were from the medium-sized municipality, 8 from the rural municipality, 13 from the urban district in Oslo West, and 10 from the urban district in Oslo East. An overview of their professional background and age is provided in Table 2. In each municipality/district we aimed for a mix of professions and organisational roles to capture a breadth of perspectives across home care, rehabilitation, social care, and allocation services.

**Table 2:** Characteristics of the participants

Category	Number
Healthcare professionals (*N* = 38)	
Nurses	9
Healthcare workers	9
Occupational therapists	7
Physical therapists	6
Social workers	4
Case managers	2
Medical doctor	1
Age intervals	
20–25 years	3
26–30 years	8
31–40 years	9
41–50 years	8
51–60 years	6
61–65 years	4

### Data collection

A total of eight focus group interviews were conducted, with two in each of the four participating municipalities/districts, involving a mix of professionals from various backgrounds and municipal sectors. This mixed-professional composition was deliberate to elicit varied and potentially conflicting perspectives across frontline providers. Employees were recruited with the assistance of a key contact person in each setting. These contacts (e.g. service managers or coordinators) helped identify eligible frontline staff and distributed written information about the study and the invitation to participate. Participation was voluntary, and staff who wished to take part contacted the researchers or the key contact to arrange attendance during working hours. All focus groups took place in person between April and June 2025. Two researchers collected the data: a health services researcher with a background in physiotherapy and experience from municipal healthcare, who led the interviews, and a housing researcher specialising in social policy and the welfare state, who acted as an observer. This collaboration integrated health and social science perspectives to explore how services support older adults to live safely at home, balancing close ‘inside’ engagement with appropriate analytical ‘outside’ distance [25]. Throughout data collection and analysis, the researchers discussed how their professional backgrounds and prior experiences might shape the questions asked, the interaction with participants, and the interpretation of the data, and they actively sought alternative readings to nuance the analysis and reduce bias. The mixed-professional composition stimulated cross-professional dialogue, as participants compared and sometimes contested each other’s perspectives. The participants thus represented a broad range of primary care professions and organisational roles involved in the day-to-day delivery and coordination of municipal services for older adults.

A semi-structured interview guide was refined in collaboration with a stakeholder group comprising members from all participating municipalities and districts and a reference group of individuals in strategic positions in key national organisations and authorities. The stakeholder group ensured that the questions reflected local service realities and terminology, while the reference group contributed a broader policy and system-level perspective. These groups were established as part of the larger funded project and were used here to strengthen quality and relevance. The final version of the interview guide is presented in Appendix 1.

To ensure consistency, the guide included themes such as (i) the role of services in enabling older adults to live safely at home, (ii) creating inclusive and age-friendly communities, (iii) housing adaptation and age-friendly housing in coordination with services, (iv) accessible, high-quality services with competent personnel, and (v) ensuring user safety and providing support to caregivers. The interviews were subsequently discussed with the same stakeholder and reference groups to challenge unfounded assumptions. The focus groups lasted 70–90 min. All sessions were audio-recorded, stored on the Services for Sensitive Data platform, and transcribed verbatim in accordance with privacy regulations. We were aware that professional hierarchies could shape the focus group interaction. The moderator emphasised that all experiences were equally important and actively invited quieter participants to speak and managed dominant voices.

### Data analysis

To analyse the data, the authors employed reflexive thematic analysis with a critical orientation approach, focusing on underlying structures shaping the experiences of healthcare professionals [27]. Reflexive thematic analysis was chosen because it is well suited to an interpretive, critical-realist approach and allows an iterative movement between participants’ accounts of everyday practice and theorisation of underlying mechanisms shaping frontline decision-making. The analysis began with the authors discussing the interview content in depth. The first author transcribed the interviews verbatim, re-read the transcripts while taking notes, and coded the data using NVivo qualitative data analysis software. The codes were grouped, and initial themes were developed and further discussed with the second author. As the analysis progressed, healthcare professionals’ daily dilemmas, strategies, and prioritising were identified as clear patterns, alongside opportunities for improvement. Lipsky’s street-level bureaucracy theory was then applied to guide the subsequent analysis. Finally, through a comprehensive comparison of quotes and the entire data set, nine initial themes were reassessed, refined, and combined into three main themes (Table 3).

**Table 3:** Thematic analysis

Initial Themes	Main Themes
Personal responsibility for ageing	Defining responsibility: supporting ageing preparation and managing expectations
Redefining necessary and dignified care	
Task shifting and welfare technology	
Lack of a municipal preventive culture	Cultivating a culture of prevention: early detection, independence, and rehabilitation
Need for early intervention and rehabilitation	
Managing expectations through guidance	
Housing accessibility and adaptations	Building inclusive communities: housing facilities, accessibility, and collaboration
Limited transport solutions in the outskirts	
Social inclusion and volunteer coordination	

The study is consistent with the concept of information power, considering the study aim, sample specificity, theoretical framework, quality of dialogue, and analytical approach [28]. In this study, the relatively focused aim, the specificity of the sample (frontline providers working with older adults across four contrasting municipal contexts), and the rich, multi-professional group discussions contributed to high information power. After eight focus group interviews our judgment was that sufficient information power had been reached, and that the findings were ready for discussion with the stakeholder and reference group. The manuscript is reported in line with the Standards for Reporting Qualitative Research [29].

## Results

The final analysis resulted in three main themes: (i) Defining responsibility: supporting ageing preparation and managing expectations, (ii) Cultivating a culture of prevention: early detection, independence, and rehabilitation, and (iii) Building inclusive communities: housing facilities, accessibility, and collaboration.

### Defining responsibility: supporting ageing preparation and managing expectations

A common concern among healthcare professionals was that older adults must take greater responsibility for their own ageing as future resources are unlikely to meet growing demand. In response, municipalities were described as reducing services and redefining what is considered necessary and dignified care. This shift, particularly evident in smaller municipalities, places greater responsibility on users and often creates unrealistic expectations, leaving frontline workers to mediate between users, their families, and the system. As one doctor explained:

“*We must focus a lot on managing expectations because the gap between what people expect and what we can provide is only growing. Our staff are running faster*,* their shifts are busier*,* and we’ve had to cut non-mandatory services*,* like mobile caretaker support*,* which were previously available*” (Doctor, municipality 2).

To address these resource challenges, participants pointed to task shifting and welfare technology. Task shifting, such as delegating non-medical tasks to volunteers or private actors, was seen as a way to optimise resources and allow frontline workers to focus on specialised care. Welfare technology, such as fall-detecting sensors, Global Positioning System trackers, and medicine dispensers, was described as a way to reduce in-person visits and free up resources. However, they emphasised that collaboration with volunteers and private actors, and the use of welfare technology, require clear systems and accountability, which were often lacking. Without coordination, their potential was perceived as limited.

Reduction in practical assistance was also seen as placing greater demands on relatives, who already contribute significantly and depend on municipal support to sustain their efforts and avoid burnout:

“*Relief services for relatives*,* like day centres or short-term stays in nursing homes for the older person*,* help families care for their loved ones at home longer. But vacancies are limited*,* applications are increasing*,* and stricter criteria for long-term care make the need for more relief options urgent*” (Nurse, district 2).

Beyond task shifting and welfare technology, participants described clear communication and proactive guidance as vital for managing expectations and supporting ageing in place. They highlighted the importance of accessible information from municipalities to older adults and their families, for instance through brochures, websites, and campaigns, as well as low-threshold counselling services on housing, health, and finances. Such initiatives were seen as ways to strengthen “ageing preparation” and align expectations:

“*In our municipality*,* the Volunteer Centre and the Healthy Living Centre organise a weekly event called ‘Thursday Talk*,*’ where topics like how to adapt your home and prevent illness are discussed. They’ve managed to reach many pensioners in meaningful ways through these efforts*” (Social care worker, municipality 1).

### Cultivating a culture of prevention: early detection, independence, and rehabilitation

Healthcare professionals also pointed to what they experienced as a weak preventive culture within the healthcare system. Many described being forced into “firefighting,” addressing crises rather than tackling issues early. They felt that this lack of a proactive approach led to missed opportunities for early intervention, effective rehabilitation, and long-term planning, which in turn contributed to faster health deterioration and greater dependency among older adults. As one physiotherapist explained:

“*We often talk about only doing the ‘need to’ and cutting out the ‘nice to*,*’ but that’s a dangerous mindset. Prevention might seem like a ‘nice to*,*’ but it’s actually a ‘need to.’ Early interventions*,* like using a medication dispenser before dementia progresses or preventing hip fractures*,* can help people stay independent longer. Preventing a single hip fracture*,* which costs 1.5 million kroner on average*,* makes these measures both essential and cost-effective. Focusing only on essential care risks losing the trust and connection with the population*” (Physical therapist, district 1).

Participants underlined that preventive care requires prioritisation at the municipal level and a strong preventive orientation among frontline providers. They argued that supporting ageing in place involves a shift in mindset, as well as time and resources. For example, home care staff were said to need dedicated time in their schedules, supported by clear decisions and plans, to deliver fall prevention initiatives or provide information about preventive services. Without this support, they reported that prevention efforts are easily overshadowed by acute care needs. As one home care assistant put it:

“*I know it sounds harsh*,* but giving out a brochure about preventive care—and taking the time to explain it—really isn’t part of my job as an assistant in home care. Should I go the extra mile*,* or just stick to what I’m required to do? I wish it weren’t like this*,* but there just isn’t enough time. Sure*,* it helps me*,* the person*,* and the council*,* but it’s not in the care plan*” (Nursing assistant, municipality 1).

Participants also described the need for clear, structured initiatives tailored to the needs of older adults across the continuum of care, such as home visits, fall prevention programmes, and expanded rehabilitation services. These were seen as ways to identify risks early and support independence, provided that frontline workers receive appropriate training, resources, and time. Empowering older adults and their families to improve independence literacy and navigate the system was likewise regarded as important. As one occupational therapist said:

“*Prevention has been cut back in recent years*,* and as a rehab team*,* we’re rarely allowed to carry out preventive home visits. The focus is now on cutting home care*,* stepping in only during crises*,* and ensuring staff cope*,* while users are left to manage with minimal dignity*” (Occupational therapist, district 1).

### Building inclusive communities: housing facilities, accessibility, and collaboration

Healthcare professionals raised concerns about the lack of suitable housing for older adults, particularly in rural areas, where accessible and affordable options were described as limited. They stressed the need for care homes with services and shared housing solutions that older adults can access before their needs become critical, enabling them to live independently and maintain quality of life for longer. They also highlighted the importance of practical assistance to adapt homes early, such as removing thresholds, modifying bathrooms, or installing assistive technology. Without such measures, participants felt that many older adults risk unnecessary hospitalisations or premature moves to institutional care:

“*We see a huge need for more care home facilities—accessible*,* single-level homes that offer social activities*,* spaces for interaction*,* and some assistance. These facilities help older adults live independently for longer*,* but with two to four-year waiting lists*,* many end up in nursing homes before getting a place*” (Case manager, district 2).

Transport was another concern. Participants described it as a “lifeline” for older adults, particularly those in rural areas or with limited mobility. Unreliable or limited transport was seen as leaving older adults isolated and unable to access essential services or social activities, with negative consequences for health and well-being. Frontline workers expressed frustration at the lack of transport solutions, and several called for municipal investments in more equitable options, such as door-to-door services:

“*There’s a need for better transport options*,* like a community-run minibus service. It could pick people up from their homes and take them to activities or even for a drive around the municipality. Other places have this*,* and it works well. Providing this kind of service would be fantastic and make a big difference for older adults*,* helping to prevent loneliness and isolation*” (Healthcare worker, municipality 2).

Participants also emphasised the importance of creating opportunities for older adults to engage in social activities and access community spaces, as loneliness and isolation were seen as persistent challenges. While many activities exist, including those offered by volunteer organisations, professionals noted that awareness of these opportunities is often limited. They called for better promotion and coordination of services, including systematised volunteer recruitment and stronger collaboration with municipalities. The absence of a more centralised approach was described as making it difficult to connect isolated older adults with available activities. As one senior advisor summarised it:

“*We have plenty of activities and services*,* but people keep asking: Where can we find the information? We lack proper channels to share this information. At the same time*,* there’s little structure in connecting volunteers with lonely older adults*,* and there aren’t enough volunteers to meet the demand either*” (Senior advisor, municipality 1).

## Discussion

The results illustrate what participating frontline providers regard as essential for safe and dignified ageing in place: emphasising personal responsibility combined with supporting preparation for ageing and managing expectations, fostering a culture of prevention with a focus on early detection and rehabilitation, and building inclusive communities prioritising accessible housing, reliable transport, and social engagement. Together, these themes highlight the need for a holistic, collaborative approach to help older adults maintain independence and quality of life despite limited resources and a growing ageing population. They also underscore the central role of community-based primary care in coordinating medical, rehabilitative, and social support for older adults living at home. As street-level practitioners operating at the interface between policy and everyday life, frontline providers offer unique insight into how ageing-in-place strategies are translated into concrete services and trade-offs in practice [14, 15]. Our study adds a street-level perspective on how frontline providers in community-based primary care interpret and negotiate ageing-in-place policies and competing priorities in everyday decision-making.

Frontline providers emphasised the importance of clear responsibility and realistic expectations for ageing safely at home yet noted that responsibilities are increasingly shifted onto older adults and families, widening the gap between expectations and available long-term care. This increased reliance on families underscores the need to consider caregiver burden when designing prioritisation strategies, as reduced support can exacerbate stress and limit relatives’ ability to help older adults age safely at home [30]. Long-term care spans prevention, treatment, rehabilitation, and palliative care across home, assisted living, and institutional settings [31]. With an ageing population and more people living with complex needs, municipal healthcare services are under increasing strain [16, 32–34], while care is shifting from hospitals to the community [35]. In this context, frontline providers must prioritise under tight constraints [14], highlighting the need for clear guidance that aligns roles and prioritisation with available resources. For primary care teams, this means balancing immediate clinical needs, rehabilitation potential, carer burden, and social vulnerability within short encounters and fragmented service pathways. In practice, acute needs often trump expected long-term benefit, pushing prevention aside. In addition, cost-effectiveness may favour technology and task shifting, while autonomy (for example, the right to live at home) can conflict with carer sustainability. These tensions may be mediated daily by frontline discretion [15] and need to be addressed explicitly in primary care planning and prioritisation tools.

Municipal strategies that emphasise greater self-responsibility and family support reinforce these ambiguities. Although prioritisation criteria for municipal health services —benefit, cost-effectiveness, severity and autonomy —formally exist [11, 12], their application is often ambiguous [13]. Decision-making spans frontline services, municipal administrations, and national authorities [11], within a framework of statutory municipal autonomy that permits substantial variation, both appropriate and inequitable [31]. As Blix and Ågotnes (2022) note, the rhetoric of “successful ageing” is easier to communicate than service downscaling, explaining the observed shift of responsibility to older people and families [36]. In line with Lipsky, our findings reflect how ambiguous criteria are translated into local routines through frontline discretion, producing variation that may at once be tailored and inequitable [14]. For primary care, this points to the need for clearer, locally adapted prioritisation criteria that explicitly consider caregiver burden and distribution of responsibility between services, older adults, and families.

Against this backdrop, frontline providers reported growing reliance on welfare technology to manage scarce resources, mirroring policy trends [7, 8]. While they acknowledged benefits, they remained ambivalent due to mixed implementation experiences [37–39]. Managers and suppliers tend to focus on efficiency, standardisation, and measurable outputs, whereas frontline providers emphasise care ethics, using technology selectively to prevent loneliness, insecurity, and digital exclusion [40]. Frontline providers saw clear potential, but only with competent use and systematic implementation. A Swedish study shows that prioritisation criteria like cost-effectiveness and autonomy may conflict, creating ambiguity in practice [41]. Frontline providers also identified scope for task shifting and reported unnecessary tasks in many settings. An umbrella review supports this and highlights enabling conditions, including municipal knowledge-building, supportive systems and stable funding [42]. As a starting point, they called for simple, proactive expectation-setting and ageing preparation support. This means plain, co-produced information and easy-access consultations on housing, health and finances, to align expectations and support fair prioritisation, especially for people with low health or digital literacy [4]. Many older adults “do not know what they do not know” about future needs and cannot plan for ageing in place without such support. Preventive home visits are one example: a health economics study in Norwegian municipalities has shown promising results for older adults, including a shift from nursing homes to home-based care, about 7% fewer hospital admissions, and more than 4% lower mortality among those aged 80 and above [43]. These dynamics reflect tensions between cost-effectiveness and autonomy and underscore the need for structured decision support in primary care [11].

Across participating municipalities and districts, a cross-cutting priority was to strengthen preventive and proactive services, including rehabilitation, across the life course. In line with World Health Organization guidance, rehabilitation can improve functional outcomes, delay dependency, and reduce hospital and long-term care use [44]. Falls prevention emerged as a key domain, as falls are a major contributor to disability among older adults globally [45, 46], often associated with chronic conditions, impaired balance, muscle weakness, and frailty [47]. Evidence supports multiple interventions such as strength and balance training [46], home modifications [48], medication reviews [49], and reablement programmes [50], with favourable cost-effectiveness in many settings. Both global [49] and national guidelines [51] recommend annual fall risk screening for adults aged 65 and older, followed by comprehensive assessment and tailored interventions for those at risk. However, our frontline providers reported that competing demands in a hectic workday limit their ability to prioritise these activities, highlighting a persistent gap between policy recommendations and routine practice. This gap emerges within primary care itself, where home care and rehabilitation services are structurally oriented towards short-term, task-based care rather than systematic, proactive prevention. Addressing this requires municipal prioritisation and system-level steps, such as brief screening in routine contacts, task shifting, clear referral pathways to community exercise and home safety services, simple digital prompts, and monitoring of uptake and outcomes, so that preventive work is supported by mandates and protected time, not only by exhortations.

Beyond falls, building a preventive culture requires organisational priorities in municipalities [52]: protected time for preventive tasks in care plans, clear roles and training for frontline staff, and structured initiatives across the continuum, such as preventive home visits [43] and reablement [50]. It also requires empowering older adults and families with clear information and navigation support, and tracking uptake and outcomes to ensure accountability [53]. Linking such navigation support to the criterion of autonomy can help ensure informed choice, particularly where ‘independence literacy’ is low, and reduce inequities in access [12]. For primary care, this means integrating navigation and counselling functions into existing services, rather than treating them as optional add-ons.

Our study highlighted concerns among healthcare professionals regarding the lack of inclusivity in ageing in place, with home care services primarily focusing on medical needs. There was a strong call for greater emphasis on age-friendly and social housing options, as well as improved transport to enable participation in social activities. The World Health Organization has emphasised the importance of age-friendly cities and communities [54], yet many countries face a shortage of appropriate, affordable, and secure housing options [55, 56], a challenge that is compounded when older adults have limited financial or social resources [57, 58]. The European Union therefore prioritises housing for older adults with low incomes in its *Affordable Housing Plan* [59]. Our frontline providers highlighted that challenges related to housing are particularly pronounced in rural areas. Accessible housing and reliable door-to-door transport are prerequisites for autonomy and benefit, not ‘add-ons’, and should be considered core components of primary care strategies for ageing in place.

Frontline providers also underscored the need for early access to housing adaptations, rather than waiting until health needs become critical. This illustrates the value of proactive—rather than reactive—services in enabling older adults to live safely at home. A recent systematic review shows that delaying housing modifications or options can lead to preventable health complications, including falls, which may accelerate transitions into institutional care. Early adaptation of the home environment enables older adults to maintain independence for longer and may help reduce pressure on the healthcare system by preventing avoidable hospital admissions [60]. This is especially crucial for persons living with dementia, as moving too late in the disease trajectory can make it difficult to adjust to a new environment, increasing confusion and distress [61]. For primary care, this implies closer collaboration with housing services and systematic integration of housing assessments into care planning for older adults with increasing needs.

When older adults lack access to essential resources such as adequate finances, community support services, or reliable transport, they face an increased risk of social isolation and loneliness, both linked to poorer mental and physical health [62]. Inclusive communities thus require not only suitable housing but also access to social activities and transport. Our informants noted that although municipal programmes and voluntary organisations offer various activities, few mechanisms exist to coordinate them, limiting visibility and accessibility [63]. One proposed initiative was an activity coordinator to enhance coordination between the public and third sectors, participation, and overall community inclusivity. Such coordination can translate autonomy into everyday participation, particularly for those with limited ‘independence literacy’ [14]. From a primary care perspective, systematic collaboration with voluntary organisations and community actors can therefore be seen as part of comprehensive support for ageing in place, rather than as peripheral.

### Strengths and limitations of the study

This study has several strengths. It draws on focus group data from four contrasting municipal contexts, capturing a broad range of primary care professions and organisational roles involved in ageing in place. The interdisciplinary research team, combining health services and housing/welfare state expertise, and the involvement of stakeholder and reference groups throughout the research process, strengthened the contextual relevance and interpretive rigour of the analysis. From a critical realist perspective, this enabled us not only to describe practice, but also to hypothesise underlying mechanisms and structures shaping frontline decisions. We also sought to enhance reflexivity by explicitly discussing how our professional backgrounds, prior research, and policy engagement might influence data collection and interpretation, and by continuously challenging preliminary interpretations within the team.

The study also has limitations. As this qualitative study is context-bound, the findings should be interpreted considering the specific Norwegian municipal settings and assessed for transferability to other contexts. Participants were recruited via key contact persons in the municipalities, which may have favoured more engaged or policy-aligned staff. Focus groups may also have limited discussion of controversial or sensitive issues, particularly around resource constraints and prioritisation. Finally, as the analysis is based on professionals’ perspectives, future research should include older adults and relatives to further explore how ageing-in-place policies are enacted and experienced.

## Conclusion

Our study shows that safe and dignified ageing in place requires a holistic approach that combines clear responsibility, a preventive culture and inclusive communities. Frontline healthcare professionals experience an increasing shift of responsibility onto older adults and their families, raising caregiver burden and underscoring the need for supportive systems. Although policy documents emphasise prevention, municipal prioritisation largely centres on legally mandated medical services, leaving frontline staff limited scope to act proactively.

These findings have practical implications for municipal primary care and policymakers. There is a need for clearer guidance and tools to support fair prioritisation, protected time and resources for preventive and rehabilitative work, and selective use of technology and task shifting. In addition, coordinated efforts across housing, transport, social activities and navigation support are required to maintain independence, reduce isolation and promote more equitable ageing in place, as seen from the perspectives of frontline providers in community-based primary care.

### Implications for primary care

From a critical realist primary care perspective, our findings point to concrete structures and mechanisms that can be modified to better realise ageing-in-place policies in everyday practice. Community-based services for older adults require: (i) operational mandates and protected time for preventive work and rehabilitation within home care and allied health services; (ii) simple, locally adapted prioritisation tools to support fair and transparent allocation decisions under resource constraints; and (iii) structured collaboration between primary care, housing, transport and the voluntary sector to address social isolation and environmental barriers to ageing in place. Strengthening these elements may help primary care teams move from predominantly reactive, crisis-driven responses towards more proactive, equitable support for older adults living at home. 

## Supplementary Information

Supplementary Material 1.

## Data Availability

The datasets generated and/or analysed during the current study are not publicly available due to restrictions in the study’s data collection approval and the associated privacy regulations and cannot be shared outside the project’s research group. For questions about the study, please contact the corresponding author, Linda Aimée Hartford Kvæl (liaikv@ahus.no).
