# Supplementary material for: Frontline providers’ perspectives on ageing in place: negotiating older adults’ self-responsibility, preventive services and community support
Source: BMC Prim Care. 2026 Jun 2;27:286. doi: 10.1186/s12875-026-03404-4 (PMC13425778; doi:10.1186/s12875-026-03404-4)
Supplement: Supplementary file 1 — Supplementary Material 1. [file 12875_2026_3404_MOESM1_ESM.docx]

**Focus Group Interview Guide – Healthcare Professionals**

**Before the interview**

Briefly explain:
• The purpose and structure of the focus group
• The aim of the research
• Confidentiality and voluntary participation

Short round of introductions.

**Introductory question**

The Norwegian reform “Living safely at home” aims to enable older people to remain safely at home for as long as possible, through better planning, prevention, targeted services and better use of resources.

- In your view, what is most important for older people to be able to live safely at home?

**Age‑friendly and vibrant local communities**

- How do you view the expectation that older people should take more responsibility for planning their own old age?
- In what ways can the municipality support older people and their families in this planning?
- How would you describe your local community in terms of opportunities for participation and inclusion for older people (e.g. activities, transport, voluntary work, collaboration with other actors)?

**Housing adaptation and age‑friendly housing**

- How do you and your services contribute to making older people’s homes and housing solutions more age-friendly?
- How would you describe current and future needs for housing solutions for older people in your municipality?
- What experiences do you have with technology or digital solutions that influence how older people can live safely at home, including people with dementia?

**Accessible, high‑quality services with competent staff**

- How would you describe your experience of working in municipal health and care services, given the aim that older people should live safely at home?
- How do you perceive the quality and organisation of services (e.g. leadership, competence, collaboration and documentation) in supporting this aim?
- How do you see the balance between what older people are expected to take responsibility for themselves and what the welfare services should provide?

**Safety for users and support for family carers**

- How would you describe the accessibility and quality of services for older people in your municipality/borough?
- How are services adapted to what is important to older people themselves, including prevention, activity and a sense of belonging?
- How would you describe collaboration with and support for family carers today, and what possibilities do you see for improving this collaboration?

**Closing question**

- Is there anything else you would like to add that we have not talked about?
